# Exploring Handgrip Strength as a Cross-cultural Correlate of Body Composition and Upper Body Strength in Older Adults from Costa Rica and Kansas

**DOI:** 10.1007/s10823-023-09481-7

**Published:** 2023-07-06

**Authors:** José Moncada-Jiménez, Eva E. Dicker, Yamileth Chacón-Araya, Mariana Peralta-Brenes, José M. Briceño-Torres, Mario Villarreal-Ángeles, Mónica Salazar-Villanea, Eric D. Vidoni, Jeffery M. Burns, David K. Johnson

**Affiliations:** 1 Human Movement Sciences Research Center, University of Costa Rica, San Jose, Costa Rica; 2 School of Physical Education and Sports, University of Costa Rica, San Jose, Costa Rica; 3 Alzheimer’s Disease Research Center-East Bay, University of California, Davis, 100 N. Wiget Lane, Suite 150, Walnut Creek, CA 94598 USA; 4 Department of Psychological Sciences, Rice University, 6100 Main St, Houston, TX 77005 USA; 5 Faculty of Sports, Juarez University of the State of Durango, Durango, Mexico; 6 Psychological Research Institute, University of Costa Rica, San Jose, Costa Rica; 7 KU Alzheimer’s Disease Center, University of Kansas, 4350 Shawnee Mission Pkwy, Fairway, KS 66205 USA

**Keywords:** Aged, Hand strength, Adiposity, Bone density, Costa Rica, Kansas

## Abstract

Sarcopenia and disability in older adults are often characterized by body composition measurements; however, the gold standard of body composition measurement, dual-energy X-ray absorptiometry (DEXA), is expensive to acquire and maintain, making its use in low and middle income countries (LMIC) it out-of-reach in developing nations. Because these LMIC will bear a disproportionate amount of chronic disease burden due to global aging trends, it is important that reliable, low-cost surrogates need to be developed. Handgrip strength (HGS) is a reliable measure of disability in older adults but has not been used widely in diverse populations. This study compared HGS to multiple measurements of body composition in older adults from the US (Kansas) and a middle-income country (Costa Rica) to test if HGS is a cross-culturally appropriate predictive measure that yields reliable estimates across developed and developing nations. Percent body fat (%BF), lean tissue mass index (LTMI), appendicular lean soft tissue index (ALSTI), body fat mass index (BFMI), bone mineral density (BMD), and HGS were measured in older Costa Ricans (n = 78) and Kansans (n = 100). HGS predicted lean arm mass with equal accuracy for both samples (p ≤ 0.05 for all groups), indicating that it is a reliable, low-cost and widely available estimate of upper body lean muscle mass. Older adults from Costa Rica showed different body composition overall and HGS than controls from Kansas. Handgrip operates equivalently in the US and Mesoamerica and is a valid estimate of lean arm muscle mass as derived by the more expensive DEXA.

## Introduction

Sarcopenia is a reliable predictor of disability, frailty, and mortality, especially in the oldest-old. Sarcopenia is an age-related change in body composition, beginning in middle age (Cherin et al., 2014) where muscle mass decreases and fat mass increases. For most practicioners, body mass index **(BMI)** is their primary assessment of body composition; however, BMI does not discriminate between muscle and adipose tissue and does not directly assess regional adiposity. Sarcopenia is best appreciated by measuring lean soft tissue, body fat, and bone density. Dual-energy X-ray absorptiometry **(DEXA)** is currently considered the gold standard (Blake & Fogelman, 2010) for measuring these separate components and a superior index of sarcopenia. DEXA works by comparing and interpolating the absorption of varying energy levels of X-rays as they pass through the body to appreciate lean, fat and bone masses. Because of the high costs associated with purchasing and maintaining DEXA machines to measure sarcopenia, they are not readily available in low and middle-income countries (LMIC). Thus, multiethnic and cross-cultural validation of an alternate, low cost and reliable measure of body composition would accelerate clinical research and practice in developing nations, where population aging will have a significant impact on the world-wide burden for chronic age-related disease.

While girth indices such as waist-to-hip and waist-to-height (Flegal et al., 2009) explain malnutrition (Briend et al., 1989) and frailty (Landi et al., 2013, 2014), they may be inaccurate for predicting percent body fat (**%BF**). Other methods used to measure muscle mass include bioelectrical impedance, computed tomography, magnetic resonance imaging, urinary excretion of creatinine, anthropometric assessments, and neutron activation assessments (Morley, 2008). Body composition can also be measured with low-cost, easy-to-administer and objective measures of muscle strength like the short physical performance battery (Guralnik et al., 1994), gait speed (Buchner et al., 1996), timed get-up-and-go test (Podsiadlo & Richardson, 1991), and the stair climb power test (Bean et al., 2007).

In the current cross-cultural research we investigated if handgrip strength **(HGS)** was associated with DEXA equivalently in healthy aging Costa Ricans **(CR)** versus healthy aging Kansans **(KS)** (Vidoni et al., 2015) and if HGS added predictive value to BMI to predict DEXA values. We chose this widely accepted measure of upper body strength (indexed by dynamometer) because it shares a similar profile of association with disability (Newman et al., 2003) and mortality (Y. H. Kim et al., 2016; Sobestiansky et al., 2019) as DEXA. Furthermore, HGS has been associated with cognitive decline (McGrath et al., 2019; Rogers & Jarrott, 2008) and may also be a low-cost index to help stratify older adults at risk to lose their independence. HGS may prove to be an excellent and low-cost index of muscle mass that will add precision to traditional measures of body composition. Further, we want to test if the association between HGS and DEXA is not influenced by well-documented differences in height and body weight distribution across ethnic groups. In this context, ethnic-specific heterogeneity of risk factors for sarcopenia, disability, and frailty calls for culturally sensitive and reliable prediction of DEXA values using low-cost alternatives like HGS. Thus, HGS is a variable of great interest in this multiethnic comparison of predictors of health and disability in older adults, as it has been used primarily in the US and EU with majority white populations.

The primary purpose of this paper is to describe the efficacy of HGS as a predictive tool for health outcomes in both European Americans and Central Americans by comparing the relationship between HGS and measurements of body composition and upper body strength using the DEXA across race and gender. We hypothesize the HGS predicts DEXA values equally well in Meso-Americans as in Euro-Americans.

## Methods

### Participants

Seventy-eight CR participants (26 men and 52 women) were recruited from the Epidemiology and Development of Alzheimer's Disease Project (EDAD), the Costa Rican Gerontological Association (AGECO), and the Integral Program for Older Adults (PIAM) from the University of Costa Rica (UCR). These participants were given a measurement appointment at the Human Movement Sciences Research Center at the UCR in San José, CR (CIMOHU). In the US, a sample of 100 European Americans from KS were recruited (35 men and 65 women) and were given a measurement appointment at the Alzheimer’s Disease Center at the University of Kansas. The Scientific Ethics Committee at the UCR and the Institutional Review Board at the University of Kansas approved their respective research protocols.

### Instruments and procedures

Participants underwent body height (cm), mass (kg), and body composition assessment using standard protocols (American College of Sports Medicine, 2010, 2014; Nana et al., 2015). Body height was measured to the nearest 0.1 cm using a stadiometer. Individuals stood still with their heads in the Frankfort horizontal plane, barefoot, feet together, and the back surfaces of the calcaneus, pelvic, pectoral girdles and occipital regions in contact with the wall. Body mass was measured on a digital platform scale where individuals remained in light clothing, barefoot, feet positioned in the center of the platform, and arms next to their bodies.

In both testing sites, dual-energy X-ray absorptiometry (DEXA; Lunar Prodigy (GE Medical Systems, Madison, WI, USA) was used to determine regional and total body composition variables of fat mass, lean mass, and bone material density (**BMD**). Scans were performed according to the laboratory standard protocol following safety precautions and quality control according to international standards (Lewiecki et al., 2016). The appendicular lean soft tissue (**ALST**) in kilograms was considered equivalent to the sum of total lean soft tissue in both right and left arms and legs. Intermuscular adipose tissue-free skeletal muscle mass (**IMAT-F SMM**) was determined by the equation developed from magnetic resonance imaging scans (Kim et al., 2004) as follows: 1.19 (ALST)—1.65 ± 1.46 kg. In addition, body fat mass index (**BFMI**; total fat mass/height^2^), lean tissue mass index (**LTMI**; total lean mass/height^2^), and ALST index (**ALSTI**; ALST/height^2^) were calculated.

Handgrip strength (HGS) was measured with a hand-held dynamometer. The KS sample used a JAMAR hydraulic dynamometer (Patterson Medical, Warrenville, IL, USA) and the CR sample an electronic CAMRY, model EH-101 dynamometer (CAMRY, City of Industry, CA, USA). HGS was measured in a seated position with the elbow flexed at 90°, forearm in neutral position, and wrist between 0° and 30° dorsiflexion and between 0° and 15° ulnar deviation. Participants were encouraged to squeeze the dynamometer as hard as they could and to maintain it that way for 3–5 s. HGS was measured three times on the dominant hand. The final score was the average of the three attempts, which has been reported to provide the highest test–retest reliability (Haidar et al., 2004; Shiratori et al., 2014). HGS values were transformed to z-scores (z-score = (raw score – mean) / standard deviation) for statistical analysis given that two different dynamometer brands were used during data collection (Amaral et al., 2012).

### Statistical analysis

Statistical analysis was performed with the IBM-SPSS Statistics, version 22 (IBM Corporation, Armonk, NY, USA). Descriptive statistics are presented as mean and standard deviation (M ± SD), unless otherwise noted. Inferential analysis was performed by 2 × 2 (sample by gender) ANOVA on anthropometric, body composition and strength variables. Post-hoc analysis was completed using Tukey comparisons. Because of an observed difference between mean age in the CR and KS groups, a single factor ANOVA was conducted to assess the between group differences. Because body composition and strength change across a lifespan, age was included as a covariate in all analyses to equate the CR and KS groups on all outcomes. Adjusted Pearson correlations were then computed between HGS and anthropometric and body composition variables controlling for age and BMI.

## Results

Descriptive statistics were collected to characterize the two racial groups by body composition, and gender (Appendix Table 1). In general, the KS sample was older than the CR sample (72.84 ± 5.59 vs. 68.91 ± 4.79 yr.; *p* ≤ 0.001). The CR sample was shorter than the KS sample (158.63 ± 8.77 vs. 167.39 ± 9.72 cm; *p* ≤ 0.001), and women were shorter than men (158.54 ± 7.21 vs. 172.99 ± 8.39 cm; *p* ≤ 0.001). A significant interaction between samples and genders in body weight was found (*p* = 0.046). Post hoc analysis showed that within KS and CR samples, men were heavier than women (84.27 ± 16.15 vs. 69.05 ± 11.61 kg; *p* < 0.05). In addition, between samples, KS participants had a higher body weight than CR participants (78.60 ± 15.28 vs. 68.84 ± 13.17 kg; *p* < 0.05). No significant interactions (*p* = 0.293) or main effects between samples (*p* = 0.222) and genders (*p* = 0.511) were found on BMI.

In general, KS older adults showed higher total adiposity (29.59 ± 8.83 vs. 25.59 ± 8.40 kg; *p* = 0.001), regional arms adiposity (2.62 ± 0.93 vs. 2.30 ± 0.82 kg; *p* = 0.003), legs adiposity (9.62 ± 3.63 vs. 7.96 ± 3.22 kg; *p* = 0.001), and trunk adiposity (29.59 ± 8.83 vs. 25.59 ± 8.40 kg; *p* = 0.001) than CR older adults. Within genders, women showed higher regional arms (2.62 ± 0.79 vs. 2.20 ± 1.01 kg; *p* = 0.001) and legs (9.88 ± 3.25 vs. 7.02 ± 3.33 kg; *p* ≤ 0.001) adiposity than men. However, no significant interaction was found between samples and genders on fat arm mass (*p* = 0.058), fat leg mass (*p* = 0.814), or total fat mass (*p* = 0.173). A significant interaction was found between samples and genders on fat trunk mass (*p* = 0.042). Follow-up analysis indicated that within men, fat trunk mass was lower in the CR older adult than in the KS older adult (*p* = 0.004). Fat trunk mass was similar between KS and CR women (*p* = 0.774). In addition, men had lower %BF than women (31.24 ± 7.02 vs. 41.28 ± 5.87%; *p* ≤ 0.001), but no significant interaction was found between samples and genders on %BF (*p* = 0.182). In this study, regardless of the sample, women had higher BFMI than men (11.3 ± 3.2 vs. 8.9 ± 3.2 kg/m^2^; *p* ≤ 0.001). There was also no significant interaction found between samples and genders on BFMI (*P* = 0.264).

In this study, men showed more lean mass tissue than women (Appendix Table 1). Compared to women, men had higher lean arm mass (6.34 ± 1.60 vs. 3.72 ± 0.59 kg; *p* ≤ 0.001) and higher lean trunk mass (27.08 ± 9.05 vs. 18.95 ± 2.48 kg; *p* ≤ 0.001). Additionally, women had lower LTMI (15.0 ± 1.7 vs. 18.0 ± 1.7 kg/m^2^; *p* ≤ 0.001) and ALSTI (6.2 ± 0.8 vs. 8.1 ± 1.0 kg/m^2^; *p* ≤ 0.001) than men. No significant interactions were found between samples and genders on lean arm mass (*p* = 0.251), lean trunk mass (*p* = 0.593), LTMI (*p* = 0.527), or ALSTI (*p* = 0.668). However, regardless of gender, KS participants showed higher lean arm mass than CR participants (4.85 ± 1.51 vs. 4.34 ± 1.75 kg; *P* = 0.002). Also, regardless of gender, lean leg mass (*p* = 0.017), total lean mass (*p* = 0.030), and IMAT-F SMM (*p* = 0.025) was lower in the CR group than in the KS group. Follow-up analysis indicated that within men, lean leg mass (*p* ≤ 0.001), total lean mass (*p* ≤ 0.001), and IMAT-F SMM (*p* ≤ 0.001) was lower in the CR participants than in the KS participants, and within women, lean leg mass (*p* = 0.095), total lean mass (*p* = 0.174), and IMAT-F SMM (*p* = 0.180) was similar. Within the KS sample and within the CR sample, men had higher lean leg mass, total lean mass, and IMAT-F SMM than women (*p* ≤ 0.001 for all).

In general, KS older adults showed higher total BMD (1.16 ± 0.11 vs. 1.08 ± 0.11 g/cm^2^; *p* ≤ 0.001) than CR older adults. Additionally, regional head (2.23 ± 0.27 vs. 2.07 ± 0.30 g/cm^2^; *p* = 0.001), arms (0.83 ± 0.12 vs. 0.78 ± 0.09 g/cm^2^; *p* ≤ 0.001), legs (1.26 ± 0.18 vs. 1.14 ± 0.16 g/cm^2^; *p* ≤ 0.001), ribs (0.69 ± 0.10 vs. 0.64 ± 0.07 g/cm^2^; *p* ≤ 0.001), pelvis (1.11 ± 0.13 vs. 1.04 ± 0.12 g/cm^2^; *p* ≤ 0.001), and spine (1.08 ± 0.18 vs. 1.04 ± 0.14 g/cm^2^; *p* = 0.034) BMD were higher in KS older adults than CR older adults. For all BMD measures, men showed higher BMD than women. No significant interactions were found between samples and genders for all BMD measures (Appendix Table 1).

No significant interaction was found between samples and genders on HGS z-scores (*p* = 0.843). Men had higher HGS than women (1.01 ± 0.87 vs. -0.55 ± 0.48 z-score; *p* ≤ 0.001). Significant correlations were obtained between HGS and age (r = -0.18, 95% CI [-0.03, -0.32], *p* ≤ 0.05). HGS was also significantly associated (p < 0.001 for all) with body height, body weight, fat trunk, head BMD, legs BMD, ribs BMD, pelvis BMD, spine BMD, total BMD, lean arms mass, lean legs mass, lean mass trunk, total lean mass, and %BF, BFMI, LTMI, and ALSTI. The correlations between HGS and anthropometric and body composition variables were different between men and women from CR and KS (Appendix Table 2). HGS was significantly predictive of age in CR men (r = -0.27, 95% CI [-0.01, -0.43], *p* ≤ 0.05), CR women (r = -0.47, 95% CI [-0.23, -0.66], *p* ≤ 0.001), KS men (r = -0.44, 95% CI [-0.12, -0.67], *p* ≤ 0.01) and KS women (r = -0.28, 95% CI [-0.04, -0.49]).

**Fig. 1:**
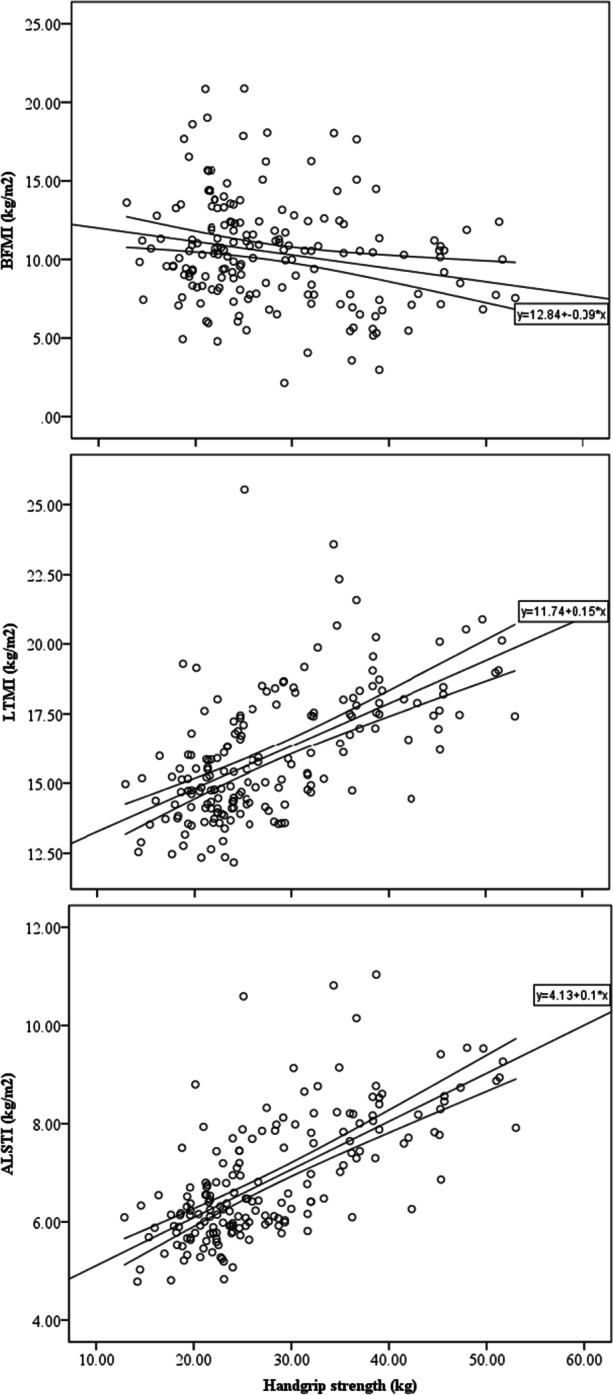
Scatterplots for the association between handgrip strength and body composition indexes in older adults (n = 178). Lines are best fit and CI_95%_

When controlled for age, the only significant correlation that extended across both samples and genders was between HGS and lean arms mass (CR men r adj = 0.36, CR women r adj = 0.48, KS men r adj = 0.64, KS women r adj = 0.42; *p* ≤ 0.05 for all). As expected, BMI was heavily correlated with a majority of anthropometric and body composition variables across samples and gender groups (Appendix Table 2). When controlled for BMI, lean arms mass (CR men r adj = 0.44, CR women r adj = 0.46, KS men r adj = 0.71, KS women r adj = 0.48; *p* ≤ 0.05 for all) and total lean mass (CR men r adj = 0.42, CR women r adj = 0.39, KS men r adj = 0.50, KS women r adj = 0.56; *p* ≤ 0.05 for all) were significantly correlated with HGS across samples and gender (Appendix Table 2 and Fig. 1).

## Discussion

Body composition measures differed between older adults from the US and CR. HGS was a dominant predictor of age and the upper body strength biomarker, lean arm mass, in both samples. Men had higher HGS than women and KS women’s biomarkers of upper body strength showed the greatest relationship to HGS. From the strong correlations between HGS and measures of body composition across sample and gender, we expected a similarly robust predictive relationship when controlled for age. However, only lean arm mass was significantly related to HGS across cultural groups and gender. ALSTI showed the potential of a subtle effect, but the relationship was unclear or lost due to its addition of lean leg mass, indicating the effect to lie with lean arm mass alone. This weakened relationship suggests that age confounded for some of the original associations seen with HGS and the biomarkers of upper body strength. These findings converge with previously published reports from international cohorts, suggesting that lean arm mass may be an indicator of overall health in aging across racial groups that have characteristically varying body composition measures.

Significant race and gender differences in adiposity were observed in the present study, a previously reported finding (Silva et al., 2010). However, the absence of differences in BMI between men and women from CR and KS supports the discussion regarding the appropriateness of this marker as a phenotypic proxy of adiposity across populations differing in race and ethnicity (Heymsfield et al., 2016; Natale & Rajagopalan, 2014). Based on the findings of this study, BMI may be a questionable measure of adiposity across racial groups.

To find that women are shorter than men in both samples, and that Caucasians are taller and heavier than Mexican and Central Americans follows established patterns since childhood, where approximately 20% of variation in body height is attributed to, but not limited, to environmental variation (e.g., nutrition, diseases) (Clark et al., 2016; Dodds et al., 2016; Heymsfield et al., 2016; Natale & Rajagopalan, 2014; Silventoinen, 2003). To further contextualize the variation in body composition and HGS across racial and ethnic groups, a thorough literature review across international cohorts is presented in Appendix B. A larger cross-cultural comparison enhances the understanding of the pragmatic application of HGS as a translation of previous methods for multiethnic populations.

## Limitations

The primary limitation experienced was the wide difference between the mean ages of the two sample groups. Age was found to have a robust effect on the relationship seen between HGS and the measures of body composition and biomarkers of upper body strength. This limitation was mitigated by controlling for age in our analyses. Another limitation in the make-up of the samples was the low number of participants. This may have impacted the power of the relationships between HGS and biomarkers of upper body strength. In the future, multiethnic comparisons should be made between large sample sizes of demographically comparable groups to amplify all subtle effects. Never-the-less these data indicate that comparing BMD between different groups also requires the development of ethnic-specific reference data. Most DEXA equipment usually reports European American reference values, which have been shown to misclassify other populations (e.g., Chinese) (Lo et al., 2016). In this study, we used the same DEXA equipment brand, software (i.e., reference standards) and calibration process; therefore, we reduced the variation between DEXA equipment brands influencing BMD scores and classification (Schousboe et al., 2014).

A source of potential error or variance also came from measuring HGS with two different dynamometer brands. Strong associations have been reported between different dynamometer brands and the JAMAR dynamometer, considered a benchmark in hand dynamometry (r > 0.77); however, the agreement between HGS was poor (Guerra & Amaral, 2009). We corrected potential bias in this measure as best as possible by z-transforming raw scores for analysis.

## Conclusion

Results of this study show that there are significant physiological differences between older US Euro-American and Meso-American adults. A comparison across nations highlights the potential of HGS to serve as an effective, universal predictor of upper body strength, and the corresponding health outcomes in older adults. Biological and environmental factors may affect aspects of physical health like muscle function, body mass, and bone mineral density, which could play a role in explaining variation in cardiovascular disease, hypertension, diabetes mellitus, asthma, and mortality across racial groups (Dodds et al., 2016; Leong et al., 2016; Silverman, 2015; Stenholm et al., 2012). Furthermore, the observed ethnic-specific heterogeneity on biologic factors and physical-related performance creates a need for culturally diverse prevention programs for older adults. While the gold standard of body composition measurement is currently DEXA, this methodology is expensive to acquire and maintain, limiting the accuracy of reference values used to predict health outcomes in low and middle income countries. HGS is a reliable, low-cost alternate to DEXA for the measurement of proxies of age-related disability across cultures. The results of this study justify further investigation into developing HGS as a standardized tool for healthcare providers for a global population.

## Figures and Tables

**Fig. 2:**
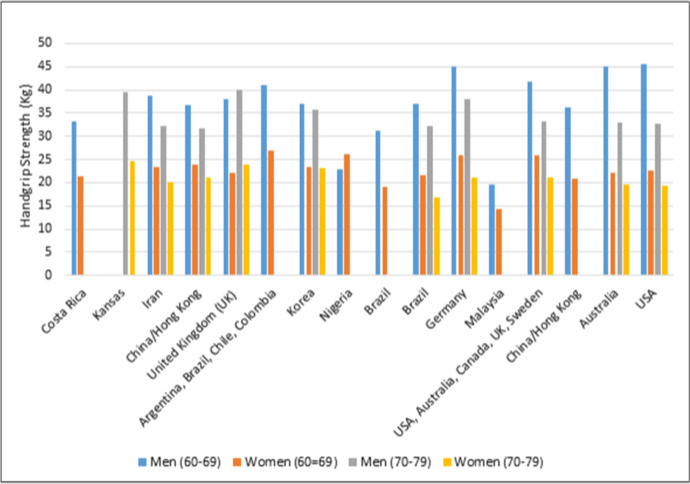
Comparative handgrip strength values (kg) acquired from previous studies in international cohorts

## Data Availability

All data and SAS codes available from the senior author upon request.

## Appendix A

Descriptive Statistics and Analysis of Body Composition and Handgrip Strength Measures Between Kansans and Costa Ricans.

**Table 1:** Descriptive statistics (Mean ± SD) for anthropometric and upper body strength of older adults from Costa Rica and Kansas (n = 178). *****

Variable	Costa Rica (n = 78)		Kansas (n = 100)	
	Men (n = 26)	Women (n = 52)	Men (n = 35)	Women (n = 65)
Age (yr.)	68.9 ± 4.5^a^	68.9 ± 5.0^a^	73.6 ± 6.4^b^	72.5 ± 5.1^b^
Body height (cm)	166.8 ± 7.3^a^	154.5 ± 6.2^b^	177.6 ± 5.9^c^	161.8 ± 6.3^d^
Body weight (kg)	75.8 ± 14.3^a^	65.3 ± 11.1^b^	90.5 ± 14.7^c^	72.1 ± 11.2^d^
BMI (kg/m^2^)	27.1 ± 3.9^a^	27.4 ± 5.0^a^	28.8 ± 5.0^a^	27.5 ± 4.2^a^
Fat mass (kg)				
• Arms	1.81 ± 0.68^a^	2.54 ± 0.78^b^	2.49 ± 1.11^c^	0.81^d^
• Legs	5.94 ± 2.51^a^	8.97 ± 3.07^b^	7.82 ± 3.66^c^	10.61 ± 3.23^d^
• Trunk	14.11 ± 5.63^a^	15.07 ± 5.71^b^	18.01 ± 6.06^c^	15.42 ± 4.75^d^
Total fat mass (kg)	22.98 ± 8.71^a^	26.90 ± 8.01^a^	29.83 ± 10.38^b^	29.64 ± 7.95^b^
BFMI (kg/m^2^)	8.2 ± 2.8^a^	11.3 ± 3.4^b^	9.4 ± 3.5^a^	11.3 ± 3.0^b^
DEXA body fat (%)	30.6 ± 7.4^a^	42.2 ± 6.3^b^	31.7 ± 6.8^a^	40.5 ± 5.4^b^
Lean mass (kg)				
• Arms	5.94 ± 2.21^a^	3.55 ± 0.53^b^	6.64 ± 0.86^c^	0.60^d^
• Legs	16.24 ± 2.37^a^	11.08 ± 1.49^b^	19.17 ± 2.53^c^	1.50^d^
• Trunk	26.98 ± 13.71^a^	18.32 ± 2.21^b^	27.15 ± 2.53^a^	19.46 ± 2.58^b^
• IMAT-F SMM	24.75 ± 4.36^a^	15.75 ± 2.24^b^	29.06 ± 3.76^c^	17.88 ± 2.41^d^
Total lean mass (kg)	49.79 ± 6.63^a^	35.65 ± 4.02^b^	57.19 ± 5.55^c^	39.10 ± 4.32^d^
LTMI (kg/m^2^)	18.8 ± 1.6^a^	15.0 ± 2.0^b^	18.2 ± 1.9^a^	14.9 ± 1.5^b^
ALSTI (kg/m^2^)	7.9 ± 1.0^a^	6.1 ± 0.9^b^	8.2 ± 1.0^a^	6.3 ± 0.7^b^
BMD (g/cm^2^)				
• Head	2.10 ± 0.28^a^	2.05 ± 0.32^b^	2.23 ± 0.29^c^	2.23 ± 0.26^d^
• Arms	0.88 ± 0.06^a^	0.73 ± 0.07^b^	0.95 ± 0.10^c^	0.77 ± 0.08^d^
• Legs	1.30 ± 0.12^a^	1.10 ± 0.11^b^	1.44 ± 0.13^c^	1.17 ± 0.10^d^
• Ribs	0.70 ± 0.08^a^	0.62 ± 0.05^b^	0.76 ± 0.08^c^	0.65 ± 0.08^d^
• Pelvis	1.12 ± 0.12^a^	1.00 ± 0.10^b^	1.20 ± 0.13^c^	1.07 ± 0.10^d^
• Spine	1.13 ± 0.20^a^	1.00 ± 0.12^b^	1.21 ± 0.19^c^	1.02 ± 0.13^d^
Total BMD (g/cm^2^)	1.17 ± 0.10^a^	1.04 ± 0.10^b^	1.30 ± 0.11^c^	1.10 ± 0.08^d^
Handgrip strength (kg)	33.2 ± 6.4^a^	21.3 ± 3.5^b^	39.6 ± 8.2^a^	24.6 ± 4.6^b^

Note: BMI = Body mass index; IMAT-F SMM = Intermuscular adipose tissue-free skeletal muscle mass; BFMI = Body fat mass index; LTMI = Lean tissue mass index; ALSTI = Appendicular lean soft tissue index; BMD = Bone mineral density

^***** a−d^ Differences in superscripts across row indicate a significant difference (*p* < 0.003)

**Table 2:** Zero order correlations of body mass index and handgrip strength and regression coefficients of hand grip after covarying age and BMI in older adult men and women from Costa Rica (n = 78) and Kansas (n = 100)

Correlation Matrix			
		Handgrip_z	
Age	Pearson's r	-0.176	*
	95% CI Upper	-0.029	
	95% CI Lower	-0.315	
Height	Pearson's r	0.627	***
	95% CI Upper	0.709	
	95% CI Lower	0.528	
Weight	Pearson's r	0.485	***
	95% CI Upper	0.591	
	95% CI Lower	0.364	
Fat Arms	Pearson's r	-0.127	
	95% CI Upper	0.021	
	95% CI Lower	-0.269	
Fat Legs	Pearson's r	-0.259	
	95% CI Upper	-0.116	
	95% CI Lower	-0.392	
Fat Trunk	Pearson's r	0.137	***
	95% CI Upper	0.278	
	95% CI Lower	-0.011	
Total Fat	Pearson's r	-0.020	
	95% CI Upper	0.127	
	95% CI Lower	-0.167	
BFMI	Pearson's r	-0.249	
	95% CI Upper	-0.105	
	95% CI Lower	-0.382	
%BF	Pearson's r	-0.486	***
	95% CI Upper	-0.365	
	95% CI Lower	-0.591	
Lean mass arms	Pearson's r	0.771	***
	95% CI Upper	0.825	
	95% CI Lower	0.704	
Lean mass legs	Pearson's r	0.752	***
	95% CI Upper	0.810	
	95% CI Lower	0.680	
Lean mass trunk	Pearson's r	0.441	***
	95% CI Upper	0.553	
	95% CI Lower	0.314	
Total Lean Mass	Pearson's r	0.766	***
	95% CI Upper	0.821	
	95% CI Lower	0.698	
LTMI	Pearson's r	0.608	***
	95% CI Upper	0.693	
	95% CI Lower	0.506	
ALST_I	Pearson's r	0.695	***
	95% CI Upper	0.764	
	95% CI Lower	0.610	
Head BMD	Pearson's r	0.064	***
	95% CI Upper	0.209	
	95% CI Lower	-0.084	
Arms BMD	Pearson's r	0.638	
	95% CI Upper	0.718	
	95% CI Lower	0.541	
Legs BMD	Pearson's r	0.574	***
	95% CI Upper	0.665	
	95% CI Lower	0.465	
Ribs BMD	Pearson's r	0.502	***
	95% CI Upper	0.605	
	95% CI Lower	0.383	
Pelvis BMD	Pearson's r	0.459	***
	95% CI Upper	0.568	
	95% CI Lower	0.334	
Spine BMD	Pearson's r	0.418	***
	95% CI Upper	0.533	
	95% CI Lower	0.288	
Total BMD	Pearson's r	0.502	***
	95% CI Upper	0.605	
	95% CI Lower	0.383	

*Note.* * *p* < 0.05, ** *p* < 0.01, *** *p* < 0.001

## Appendix B

Comparison of Body Composition and Measures of Handgrip Strength to Previous International Research.

To encourage comparison of body composition and strength measures across multiethnic groups, the descriptive statistics of the Costa Rican and Kansan samples were compared to a non-exhaustive list of studies (Auyeung et al., 2014; Clark et al., 2016; Coin et al., 2008; Fan et al., 2014; Jiang et al., 2015; C.-H. Kim et al., 2011; Shaw et al., 2007; Toomey et al., 2016; Xiao et al., 2017) reporting body composition values in older adults, which is presented in Table 3.

Comparing to previous literature in handgrip strength across multiethnic populations (Adedoyin et al., 2009; Bohannon et al., 2006; Budziareck et al., 2008; Crosby & Wehbé, 1994; Günther et al., 2008; Leong et al., 2016; Luna-Heredia et al., 2005; Massy-Westropp et al., 2004; Mathiowetz et al., 1985; Norman et al., 2011; Schlüssel et al., 2008; Spruit et al., 2013), both the Kansas and Costa Rican scores report in this manuscript fall within range with other countries reviewed here (Figure 2).

**Table 3:** Body composition values and comparison with previous studies. Values are means

Variable	Sample	Age group (yr.)				Reference
		60–69		70–79		
		Men	Women	Men	Women	
%BF	Costa Rica	30.7	42.2			Present study
	Kansas			31.7	40.5	Present study
	Ireland	28.1	38.3			(Toomey et al., 2016)
	Italy	26.1	36.0	26.0	36.6	(Coin et al., 2008)
	China	23.4	34.0	24.6	34.6	(Xiao et al., 2017)
	China	26.7		27.0		(Jiang et al., 2015)
	NHANES White	32.5	45.8	33.0	45.7	(Fan et al., 2014)
	NHANES Mexican	32.1	46.7	32.6	46.7	(Fan et al., 2014)
	Korea	23.7	36.0			(C. H. Kim et al., 2011)
	Australia	28.1	40.7	28.9	41.4	(Shaw et al., 2007)
BFMI(kg/m^2^)	Costa Rica	8.2	11.3			Present study
	Kansas			9.4	11.3	Present study
	Mexico	8.1	11.8	7.6	11.2	(Clark et al., 2016)
	Ireland	7.6	9.8			(Toomey et al., 2016)
	Italy	7.1	9.7	7.1	9.6	(Coin et al., 2008)
	China	5.3	7.6	5.4	7.7	(Xiao et al., 2017)
	China	6.4		6.8		(Jiang et al., 2015)
	NHANES White	9.3	13.0	9.1	12.5	(Fan et al., 2014)
	NHANES Mexican	9.1	13.7	8.7	13.2	(Fan et al., 2014)
	Korea	5.7	8.8			(C. H. Kim et al., 2011)
LTMI(kg/m^2^)	Costa Rica	17.9	15.0			Present study
	Kansas			18.2	14.9	Present study
	Mexico	17.6	14.6	17.0	14.5	(Clark et al., 2016)
	Ireland	18.6	15.0			(Toomey et al., 2016)
	Italy	20.3	16.9	19.4	16.0	(Coin et al., 2008)
	China	16.6	14.0	16.2	14.0	(Xiao et al., 2017)
	China	18.2		17.7		(Jiang et al., 2015)
	NHANES White	18.3	14.5	17.6	14.2	(Fan et al., 2014)
	NHANES Mexican	18.3	15.0	17.5	14.5	(Fan et al., 2014)
	Korea	17.9	15.2			(C. H. Kim et al., 2011)
ALSTI(kg/m^2^)	Costa Rica	8.0	6.1			Present study
	Kansas			8.2	6.3	Present study
	Mexico	7.8	6.1	7.4	5.9	(Clark et al., 2016)
	Ireland	8.9	6.7			(Toomey et al., 2016)
	China	7.2	5.8	6.9	5.6	(Xiao et al., 2017)
	China/Hong Kong	7.4	6.1	7.1	6.0	(Auyeung et al., 2014)
	NHANES White	8.0	6.0	7.5	5.8	(Fan et al., 2014)
	NHANES Mexican	7.9	6.1	7.3	5.8	(Fan et al., 2014)

Note: NHANES: The National Health and Nutrition Examination Survey 1999–2004; %BF: Body fat percentage; BFMI: Body fat mass index; LTMI: Lean tissue mass index; ALSTI: Appendicular lean soft tissue index

**Table 4:** Handgrip strength values (kg) comparison with previous studies. Values are means

Country	Age group (yr.)				Reference
	60–69		70–79		
	M	W	M	W	
Costa Rica	33.2	21.3			Present study
Kansas			39.6	24.6	Present study
Iran	38.7	23.3	32.1	20.2	(Mohammadian et al., 2014)
China/Hong Kong	36.8	23.8	31.8	21.2	(Auyeung et al., 2014)
United Kingdom (UK)	38.0	22.0	40.0	24.0	(Spruit et al., 2013)
Argentina, Brazil, Chile, Colombia	41.0	27.0			(Leong et al., 2016)
Korea	37.1	23.3	35.7	23.1	(Shim et al., 2013)
Nigeria	22.8	26.2			(Adedoyin et al., 2009)
Brazil	31.3	19.1			(Budziareck et al., 2008)
Brazil	37.0	21.7	32.1	16.8	(Schlüssel et al., 2008)
Germany	45.0	26.0	38.0	21.0	(Günther et al., 2008)
Malaysia	19.6	14.3			(Kamarul and Ahmad, 2006)
USA, Australia, Canada, UK, Sweden	41.7	25.8	33.1	21.1	(Bohannon et al., 2006)
China/Hong Kong	36.2	20.9			(Tsang, 2005)
Australia	45.0	22.0	33.0	19.5	(Massy-Westropp et al., 2004)
USA	45.5	22.5	32.8	19.4	(Mathiowetz et al., 1985)

Note: M: Men; W: Women

## Appendix C

### Handgrip Strength

Handgrip strength (HGS) is an estimate of general strength and a strong predictor for future health outcomes in aging populations (Bohannon, 2008; Bohannon et al., 2006). Previous research has shown that handgrip strength declines with age, as demonstrated in a longitudinal study in Chinese participants aged 60 and older. After 4 years of follow-up, the loss in handgrip strength in males was smaller (-0.590 to -0.985 kg) than in women (-1.150 to -1.380 kg) (Auyeung et al., 2014). In a large cohort of 4,912 Japanese participants, grip strength was longitudinally studied to determine an association with mortality (Sasaki et al., 2007). Handgrip strength is also considered a powerful predictor of cause-specific and total mortality in older disabled women and cardiometabolic risk in aging populations (Bohannon, 2008; Günther et al., 2008; Leong et al., 2015). In a cohort of 927 Taiwanese participants aged 53 and older, dominant handgrip strength showed a positive association with cardiometabolic risk factors (e.g., blood pressure, triglyceride, total cholesterol to high density cholesterol ratio, glycohemoglobin, uric acid, Framingham Risk Score, and fasting glucose) (Leong et al., 2015). Grip strength was found to be stronger predictor of all-cause and cardiovascular mortality than systolic blood pressure in a large (n = 139,691) cohort of participants from Canada, Sweden, United Arab Emirates, Argentina, Brazil, Chile, Malaysia, Poland, South Africa, Turkey, China, Colombia, Iran, Bangladesh, India, Pakistan, and Zimbabwe (Leong et al., 2015). If handgrip strength correlated with the biomarkers for upper body strength, it could serve as a potentially more accessible alternative to using DEXA to predict health outcomes in older adults across ethno-racial populations.

### Dual Energy X-Ray Absorptiometry

Dual-energy x-ray absorptiometry (DEXA) is considered to be the gold-standard for body composition assessment. It has been proven highly reliable (Nana et al., 2015); however, due to its high cost, such equipment was unavailable to low-income and middle-income countries until recently (Treviño-Aguirre et al., 2014). While several proxy adiposity measures and %BF prediction equations have been validated using DEXA as the criterion measure, under and overestimations have been also reported (Carpio-Rivera et al., 2016; Chambers et al., 2014). Research has shown that the association between BMI and %BF varies among different ethnic groups (Deurenberg et al., 1998) and that girth measures are inaccurate for predicting %BF in individuals (Flegal et al., 2009). Biased conclusions can be drawn from violating body composition assumptions that are not constant across ethnic groups (Deurenberg & Deurenberg-Yap, 2001). While accurate and reliable in predominately white populations, this technology does not serve as an accessible means of measurement in developing countries, and therefore has not been validated as a universal measure across racial and ethnic groups.

### Muscle Strength

A relationship between handgrip strength and muscle strength can establish handgrip strength as an accessible alternative to DEXA can be used to predict disability in older adults. Differences in aging and rates of disability in older adults can be traced to biomarkers of upper body strength. Skeletal muscle function is a key component of healthy aging and disease prevention. Muscular strength and increased muscle mass are positively related to overall health (Ruiz et al., 2008). Reduced muscle mass and poor muscle strength have been related to increased mortality through its association with increased disability (Leong et al., 2015). A large proportion of total-body skeletal muscle is found in the extremities, and a large proportion of extremity lean soft tissue is skeletal muscle (J. Kim et al., 2002). The appendicular lean soft tissue index (ALSTI) in kilograms is considered equivalent to the sum of total lean soft tissue in both right and left arms and legs, while lean tissue mass index (LMTI) is the quotient of lean mass and square of height. Lean mass measurements such as ALSTI and LMTI are strong biomarkers of upper body strength and can be used as a predictive tool in assessing overall health outcomes in older adults.

### Body Fat

Previous research shows differences in body weight and fat distribution in diverse populations and may suggest cross-cultural differences in health outcomes in aging that require a translation of current methods. Body mass and fat can also act as a predictive tool for health and mortality outcomes in an aging population. Research in a cohort of 49,476 women and 4,944 men indicated that older adults aged more than 63 years with low body mass index (BMI = body weight in kg/body height in m^2^) and increased body fat percentage (%BF) showed increased mortality (Padwal et al., 2016). Differences in adiposity content and distribution have been reported among different ethnic groups (Gasevic et al., 2015; Haldar et al., 2015; Wells, 2012). Evidence suggests that Asian groups have a greater predisposition towards adiposity at higher BMI than Caucasians (Haldar et al., 2015). (Gasevic et al., 2015) reported varying rates of higher obesity prevalence in African Americans and Hispanics living in the United States compared to Caucasians.

### Bone Mineral Density

Bone loss or the reduction in bone mineral density (BMD), a biomarker of upper body strength, has been associated with increased morbidity and mortality in older adults (Edwards et al., 2015). Reductions in BMD and fracture risk have been clearly described in the literature (Jiang et al., 2015). Bone fractures related to osteopenia and osteoporosis are common in older adults and hip fractures have been linked to mortality (Danielson & Zamulko, 2015). In South Korea, 67.7% of women aged 65 years and older and 33.5% of men aged 75 years and older are at high risk of bone fractures (J. W. Kim et al., 2014). Increased bone fracture risk was observed in a sample of 3,301 men from six sites across the United States (Birmingham, AL; Minneapolis, MN; Palo Alto, CA; Pittsburgh, PA; Portland, OR; and San Diego, CA) (Chalhoub et al., 2016). However, ethnic and racial differences exist in bone fracture epidemiology. For instance, longitudinal data on Hispanics living in the United States have shown little to no change in hip fracture incidence compared to white women and men, and Black Americans and Asian Americans showed no significant decline in hip fracture incidence over 10 years of study (Wright et al., 2012). Previous data suggests there are some differences in these biomarkers across a multiethnic population, raising the question of whether the same long-term health effects are seen in aging.

## References

[CR1] Adedoyin RA, Ogundapo FA, Mbada CE, Adekanla BA, Johnson OE, Onigbinde TA, Emechete AAI (2009). Reference Values for Handgrip Strength Among Healthy Adults in Nigeria. Hong Kong Physiotherapy Journal.

[CR2] Amaral JF, Mancini M, Novo Júnior JM (2012). Comparison of three hand dynamometers in relation to the accuracy and precision of the measurements. Brazilian Journal of Physical Therapy.

[CR3] American College of Sports Medicine. (2010). *ACSM’s health-related physical fitness assessment manual* (3rd ed.). Wolters Kluwer, Lippincott Williams & Wilkins.

[CR4] American College of Sports Medicine. (2014). *ACSM´s Guidelines for Exercise Testing and Prescription* (9th ed.). Wolters Kluwer, Lippincott Williams & Wilkins.

[CR5] Auyeung TW, Lee SWJ, Leung J, Kwok T, Woo J (2014). Age-associated decline of muscle mass, grip strength and gait speed: A 4-year longitudinal study of 3018 community-dwelling older Chinese: Longitudinal decline of muscle mass and function. Geriatrics & Gerontology International.

[CR6] Bean JF, Kiely DK, LaRose S, Alian J, Frontera WR (2007). Is Stair Climb Power a Clinically Relevant Measure of Leg Power Impairments in At-Risk Older Adults?. Archives of Physical Medicine and Rehabilitation.

[CR7] Blake GM, Fogelman I (2010). An Update on Dual-Energy X-Ray Absorptiometry. Seminars in Nuclear Medicine.

[CR8] Bohannon RW (2008). Hand-Grip Dynamometry Predicts Future Outcomes in Aging Adults. Journal of Geriatric Physical Therapy.

[CR9] Bohannon RW, Peolsson A, Massy-Westropp N, Desrosiers J, Bear-Lehman J (2006). Reference values for adult grip strength measured with a Jamar dynamometer: A descriptive meta-analysis. Physiotherapy.

[CR10] Bosy-Westphal A, Müller MJ (2015). Identification of skeletal muscle mass depletion across age and BMI groups in health and disease—There is need for a unified definition. International Journal of Obesity.

[CR11] Briend A, Garenne M, Maire B, Fontaine O, Dieng K (1989). Nutritional status, age and survival: The muscle mass hypothesis. European Journal of Clinical Nutrition.

[CR12] Buchner DM, Larson EB, Wagner EH, Koepsell TD, De Lateur BJ (1996). Evidence for a Non-linear Relationship between Leg Strength and Gait Speed. Age and Ageing.

[CR13] Budziareck MB, Pureza Duarte RR, Barbosa-Silva MCG (2008). Reference values and determinants for handgrip strength in healthy subjects. Clinical Nutrition.

[CR14] Carpio-Rivera E, Hernández-Elizondo J, Salicetti-Fonseca A, Solera-Herrera A, Moncada-Jiménez J (2016). Predictive validity of the body adiposity index in costa rican students: Body Adiposity Index in Costa Ricans. American Journal of Human Biology.

[CR15] Chalhoub D, Orwoll ES, Cawthon PM, Ensrud KE, Boudreau R, Greenspan S, Newman AB, Zmuda J, Bauer D, Cummings S, Cauley JA (2016). Areal and volumetric bone mineral density and risk of multiple types of fracture in older men. Bone.

[CR16] Chambers AJ, Parise E, Mccrory JL, Cham R (2014). A comparison of prediction equations for the estimation of body fat percentage in non-obese and obese older Caucasian adults in the United States. The Journal of Nutrition, Health & Aging.

[CR17] Cheng Q, Zhu YX, Zhang MX, Li LH, Du PY, Zhu MH (2012). Age and sex effects on the association between body composition and bone mineral density in healthy Chinese men and women. Menopause.

[CR18] Cherin P, Voronska E, Fraoucene N, de Jaeger C (2014). Prevalence of sarcopenia among healthy ambulatory subjects: The sarcopenia begins from 45 years. Aging Clinical and Experimental Research.

[CR19] Clark P, Denova-Gutiérrez E, Ambrosi R, Szulc P, Rivas-Ruiz R, Salmerón J (2016). Reference Values of Total Lean Mass, Appendicular Lean Mass, and Fat Mass Measured with Dual-Energy X-ray Absorptiometry in a Healthy Mexican Population. Calcified Tissue International.

[CR20] Coin A, Sergi G, Minicuci N, Giannini S, Barbiero E, Manzato E, Pedrazzoni M, Minisola S, Rossini M, Del Puente A, Zamboni M, Inelmen EM, Enzi G (2008). Fat-free mass and fat mass reference values by dual-energy X-ray absorptiometry (DEXA) in a 20–80 year-old Italian population. Clinical Nutrition.

[CR21] Crosby CA, Wehbé MA (1994). Hand strength: Normative values. The Journal of Hand Surgery.

[CR22] Danielson, L., & Zamulko, A. (2015). Osteoporosis: A Review. *South Dakota Medicine: The Journal of the South Dakota State Medical Association*, *68*(11), 503–505, 507–509. 26689033

[CR23] Deurenberg P, Deurenberg-Yap M (2001). Differences in body-composition assumptions across ethnic groups: Practical consequences. Current Opinion in Clinical Nutrition and Metabolic Care.

[CR24] Deurenberg P, Yap M, van Staveren W (1998). Body mass index and percent body fat: A meta analysis among different ethnic groups. International Journal of Obesity.

[CR25] Dodds RM, Syddall HE, Cooper R, Kuh D, Cooper C, Sayer AA (2016). Global variation in grip strength: A systematic review and meta-analysis of normative data. Age and Ageing.

[CR26] Edwards MH, Dennison EM, Aihie Sayer A, Fielding R, Cooper C (2015). Osteoporosis and sarcopenia in older age. Bone.

[CR27] Fan B, Shepherd JA, Levine MA, Steinberg D, Wacker W, Barden HS, Ergun D, Wu XP (2014). National Health and Nutrition Examination Survey Whole-Body Dual-Energy X-Ray Absorptiometry Reference Data for GE Lunar Systems. Journal of Clinical Densitometry.

[CR28] Flegal KM, Shepherd JA, Looker AC, Graubard BI, Borrud LG, Ogden CL, Harris TB, Everhart JE, Schenker N (2009). Comparisons of percentage body fat, body mass index, waist circumference, and waist-stature ratio in adults. The American Journal of Clinical Nutrition.

[CR29] Gasevic D, Ross ES, Lear SA (2015). Ethnic Differences in Cardiovascular Disease Risk Factors: A Systematic Review of North American Evidence. Canadian Journal of Cardiology.

[CR30] Guerra RS, Amaral TF (2009). Comparison of hand dynamometers in elderly people. The Journal of Nutrition, Health & Aging.

[CR31] Günther CM, Bürger A, Rickert M, Crispin A, Schulz CU (2008). Grip Strength in Healthy Caucasian Adults: Reference Values. The Journal of Hand Surgery.

[CR32] Guralnik JM, Simonsick EM, Ferrucci L, Glynn RJ, Berkman LF, Blazer DG, Scherr PA, Wallace RB (1994). A Short Physical Performance Battery Assessing Lower Extremity Function: Association With Self-Reported Disability and Prediction of Mortality and Nursing Home Admission. Journal of Gerontology.

[CR33] Haidar SG, Kumar D, Bassi RS, Deshmukh SC (2004). Average versus Maximum Grip Strength: Which is more Consistent?. Journal of Hand Surgery.

[CR34] Haldar, S., Chia, S. C., & Henry, C. J. (2015). Body Composition in Asians and Caucasians. In *Advances in Food and Nutrition Research* (75, 97–154). Elsevier. 10.1016/bs.afnr.2015.07.001 10.1016/bs.afnr.2015.07.001 26319906

[CR35] Heymsfield SB, Peterson CM, Thomas DM, Heo M, Schuna JM (2016). Why are there race/ethnic differences in adult body mass index-adiposity relationships? A quantitative critical review: BMI and race/ethnicity. Obesity Reviews.

[CR36] Jiang Y, Zhang Y, Jin M, Gu Z, Pei Y, Meng P (2015). Aged-Related Changes in Body Composition and Association between Body Composition with Bone Mass Density by Body Mass Index in Chinese Han Men over 50-year-old. PLOS ONE.

[CR37] Kamarul, T., & Ahmad, T. S. (2006). Hand grip strength in the adult Malaysian population. *Journal of Orthopaedic Surgery, 14*(2), 172–177. https://journals.sagepub.com/doi/pdf/10.1177/230949900601400213 10.1177/230949900601400213 16914783

[CR38] Kanis JA, Odén A, McCloskey EV, Johansson H, Wahl DA, Cooper C, on behalf of the IOF Working Group on Epidemiology and Quality of Life (2012). A systematic review of hip fracture incidence and probability of fracture worldwide. Osteoporosis International.

[CR39] Kim J, Wang Z, Heymsfield SB, Baumgartner RN, Gallagher D (2002). Total-body skeletal muscle mass: Estimation by a new dual-energy X-ray absorptiometry method. The American Journal of Clinical Nutrition.

[CR40] Kim, J., Heshka, S., Gallagher, D., Kotler, D. P., Mayer, L., Albu, J., Shen, W., Freda, P. U., & Heymsfield, S. B. (2004). Intermuscular adipose tissue-free skeletal muscle mass: estimation by dual-energy X-ray absorptiometry in adults. *Journal of Applied Physiology, 91*(2), 655–660. 10.1152/japplphysiol.00260.2004 10.1152/japplphysiol.00260.2004 15090482

[CR41] Kim C-H, Chung S, Kim H, Park J-H, Park S-H, Ji JW, Han S-W, Lee J-C, Kim JH, Park YB, Nam H-S, Kim C (2011). Norm references of fat-free mass index and fat mass index and subtypes of obesity based on the combined FFMI–%BF indices in the Korean adults aged 18–89yr. Obesity Research & Clinical Practice.

[CR42] Kim JW, Jeon Y-J, Baek D-H, Kim TN, Chang JS (2014). Percentage of the population at high risk of osteoporotic fracture in South Korea: Analysis of the 2010 Fifth Korean National Health and Nutrition Examination survey data. Osteoporosis International.

[CR43] Kim YH, Kim K-I, Paik N-J, Kim K-W, Jang HC, Lim J-Y (2016). Muscle strength: A better index of low physical performance than muscle mass in older adults. Geriatrics & Gerontology International.

[CR44] Landi F, Liperoti R, Onder G (2013). The usefulness of anthropometric measures. European Journal of Nutrition.

[CR45] Landi, F., Onder, G., Russo, A., Liperoti, R., Tosato, M., Martone, A. M., Capoluongo, E., & Bernabei, R. (2014). Calf circumference, frailty and physical performance among older adults living in the community. *Clinical Nutrition*, *33*(3), 539–544. Scopus. 10.1016/j.clnu.2013.07.013 10.1016/j.clnu.2013.07.013 23948128

[CR46] Leong, D. P., Teo, K. K., Rangarajan, S., Lopez-Jaramillo, P., Avezum, A., Orlandini, A., Seron, P., Ahmed, S. H., Rosengren, A., Kelishadi, R., Rahman, O., Swaminathan, S., Iqbal, R., Gupta, R., Lear, S. A., Oguz, A., Yusoff, K., Zatonska, K., Chifamba, J., … Yusuf, S. (2015). Prognostic value of grip strength: Findings from the Prospective Urban Rural Epidemiology (PURE) study. *The Lancet*, *386*(9990), 266–273. 10.1016/S0140-6736(14)62000-6 10.1016/S0140-6736(14)62000-6 25982160

[CR47] Leong, D. P., Teo, K. K., Rangarajan, S., Kutty, V. R., Lanas, F., Hui, C., Quanyong, X., Zhenzhen, Q., Jinhua, T., Noorhassim, I., AlHabib, K. F., Moss, S. J., Rosengren, A., Akalin, A. A., Rahman, O., Chifamba, J., Orlandini, A., Kumar, R., Yeates, K., … Yusuf, S. (2016). Reference ranges of handgrip strength from 125,462 healthy adults in 21 countries: A prospective urban rural epidemiologic (PURE) study. *Journal of Cachexia, Sarcopenia and Muscle*, *7*(5), 535–546. 10.1002/jcsm.12112 10.1002/jcsm.12112 PMC4833755 27104109

[CR48] Lewiecki, E. M., Binkley, N., Morgan, S. L., Shuhart, C. R., Camargos, B. M., Carey, J. J., Gordon, C. M., Jankowski, L. G., Lee, J-K., & Leslie W. D. (2016). Best practices for dual-energy X-Ray absorptiometry measurement and reporting: International society for clinical densitometry guidance. *Journal of Clinical Densitometry,* *19*(2), 127–140. 10.1016/j.jocd.2016.03.003 10.1016/j.jocd.2016.03.003 27020004

[CR49] Lo JC, Kim S, Chandra M, Ettinger B (2016). Applying ethnic-specific bone mineral density T-scores to Chinese women in the USA. Osteoporosis International.

[CR50] Luna-Heredia E, Martín-Peña G, Ruiz-Galiana J (2005). Handgrip dynamometry in healthy adults. Clinical Nutrition.

[CR51] Massy-Westropp N, Rankin W, Ahern M, Krishnan J, Hearn TC (2004). Measuring grip strength in normal adults: Reference ranges and a comparison of electronic and hydraulic instruments. The Journal of Hand Surgery.

[CR52] Mathiowetz V, Kashman N, Volland G, Weber K, Dowe M, Rogers S (1985). Grip and pinch strength: Normative data for adults. Archives of Physical Medicine and Rehabilitation.

[CR53] McGrath R, Robinson-Lane SG, Cook S, Clark BC, Herrmann S, O’Connor ML, Hackney KJ (2019). Handgrip Strength Is Associated with Poorer Cognitive Functioning in Aging Americans. Journal of Alzheimer’s Disease.

[CR54] Mohammadian, M., Choobineh, A., Haghdoost, A., & Hasheminejad, N. (2014). Normative data of grip and pinch strengths in healthy adults of Iranian population. *Iran Journal of Public Health, 43*(8), 1113–1122. https://www.ncbi.nlm.nih.gov/pmc/articles/PMC4411908/PMC4411908 25927041

[CR55] Morley JE (2008). Sarcopenia: Diagnosis and treatment. The Journal of Nutrition Health and Aging.

[CR56] Nana A, Slater GJ, Stewart AD, Burke LM (2015). Methodology Review: Using Dual-Energy X-Ray Absorptiometry (DXA) for the Assessment of Body Composition in Athletes and Active People. International Journal of Sport Nutrition and Exercise Metabolism.

[CR57] Natale V, Rajagopalan A (2014). Worldwide variation in human growth and the World Health Organization growth standards: A systematic review. BMJ Open.

[CR58] Newman AB, Haggerty CL, Goodpaster B, Harris T, Kritchevsky S, Nevitt M, Miles TP, Visser M, Aging TH, Compositi B (2003). Strength and Muscle Quality in a Well-Functioning Cohort of Older Adults: The Health, Aging and Body Composition Study. Journal of the American Geriatrics Society.

[CR59] Norman K, Stobäus N, Gonzalez MC, Schulzke J-D, Pirlich M (2011). Hand grip strength: Outcome predictor and marker of nutritional status. Clinical Nutrition.

[CR60] Padwal R, Leslie WD, Lix LM, Majumdar SR (2016). Relationship Among Body Fat Percentage, Body Mass Index, and All-Cause Mortality: A Cohort Study. Annals of Internal Medicine.

[CR61] Podsiadlo D, Richardson S (1991). The Timed “Up & Go”: A Test of Basic Functional Mobility for Frail Elderly Persons. Journal of the American Geriatrics Society.

[CR62] Rantanen T, Volpato S, Luigi Ferrucci M, EinoHeikkinen M, Fried LP, Guralnik JM (2003). Handgrip Strength and Cause-Specific and Total Mortality in Older Disabled Women: Exploring the Mechanism. Journal of the American Geriatrics Society.

[CR63] Rantanen T, Masaki K, He Q, Ross GW, Willcox BJ, White L (2012). Midlife muscle strength and human longevity up to age 100 years: A 44-year prospective study among a decedent cohort. Age.

[CR64] Redmond J, Jarjou LMA, Zhou B, Prentice A, Schoenmakers I (2014). Ethnic differences in calcium, phosphate and bone metabolism. Proceedings of the Nutrition Society.

[CR65] Rogers SD, Jarrott SE (2008). Cognitive Impairment and Effects on Upper Body Strength of Adults with Dementia. Journal of Aging and Physical Activity.

[CR66] Ruiz JR, Sui X, Lobelo F, Morrow JR, Jackson AW, Sjostrom M, Blair SN (2008). Association between muscular strength and mortality in men: Prospective cohort study. BMJ.

[CR67] Sasaki H, Kasagi F, Yamada M, Fujita S (2007). Grip Strength Predicts Cause-Specific Mortality in Middle-Aged and Elderly Persons. The American Journal of Medicine.

[CR68] Schlüssel MM, dos Anjos LA, de Vasconcellos MTL, Kac G (2008). Reference values of handgrip dynamometry of healthy adults: A population-based study. Clinical Nutrition.

[CR69] Schousboe JT, Tanner SB, Leslie WD (2014). Definition of Osteoporosis by Bone Density Criteria in Men: Effect of Using Female Instead of Male Young Reference Data Depends on Skeletal Site and Densitometer Manufacturer. Journal of Clinical Densitometry.

[CR70] Shaw KA, Srikanth VK, Fryer JL, Blizzard L, Dwyer T, Venn AJ (2007). Dual energy X-ray absorptiometry body composition and aging in a population-based older cohort. International Journal of Obesity.

[CR71] Shim, J. H., Roh, S. Y., Kim, J. S., Lee, D. C., Ki, S. H., Yang, J. W., Jeon, M. K., & Lee, S. M. (2013). Normative measurements of grip and pinch strengths of 21st century Korean population. *Archives of Plastic Surgery, 40*(1), 52–56. 10.5999/aps.2013.40.1.52 10.5999/aps.2013.40.1.52 PMC3556534 23362480

[CR72] Shiratori, A. P., Iop, R. da R., Borges Júnior, N. G., Domenech, S. C., & Gevaerd, M. da S. (2014). Evaluation protocols of hand grip strength in individuals with rheumatoid arthritis: A systematic review. *Revista Brasileira De Reumatologia*, *54*(2), 140–147 24878861

[CR73] Silva AM, Shen W, Heo M, Gallagher D, Wang Z, Sardinha LB, Heymsfield SB (2010). Ethnicity-related skeletal muscle differences across the lifespan. American Journal of Human Biology.

[CR74] Silventoinen K (2003). Determinants of Variation in Adult Body Height. Journal of Biosocial Science.

[CR75] Silverman IW (2015). Age as a moderator of the secular trend for grip strength in Canada and the United States. Annals of Human Biology.

[CR76] Sobestiansky S, Michaelsson K, Cederholm T (2019). Sarcopenia prevalence and associations with mortality and hospitalisation by various sarcopenia definitions in 85–89 year old community-dwelling men: A report from the ULSAM study. BMC Geriatrics.

[CR77] Spruit MA, Sillen MJH, Groenen MTJ, Wouters EFM, Franssen FME (2013). New Normative Values for Handgrip Strength: Results From the UK Biobank. Journal of the American Medical Directors Association.

[CR78] Stenholm S, Tiainen K, Rantanen T, Sainio P, Heliövaara M, Impivaara O, Koskinen S (2012). Long-Term Determinants of Muscle Strength Decline: Prospective Evidence from the 22-Year Mini-Finland Follow-Up Survey. Journal of the American Geriatrics Society.

[CR79] Toomey C, Leahy S, McCreesh K, Coote S, Jakeman P (2016). The body composition phenotype of Irish adults aged 18–81 years. Irish Journal of Medical Science (1971 -).

[CR80] Treviño-Aguirre E, López-Teros T, Gutiérrez-Robledo L, Vandewoude M, Pérez-Zepeda M (2014). Availability and use of dual energy X-ray absorptiometry (DXA) and bio-impedance analysis (BIA) for the evaluation of sarcopenia by Belgian and Latin American geriatricians. Journal of Cachexia, Sarcopenia and Muscle.

[CR81] Tsang, R. C. C. (2005). Reference values fo 6-minute walk test and hand-grip strength in healthy Hong Kong Chinese adults. *Hong Kong Physiotherapy Journal, 23*(1), 6–12. 10.1016/S1013-7025(09)70053-3

[CR82] Vidoni ED, Johnson DK, Morris JK, Van Sciver A, Greer CS, Billinger SA, Donnelly JE, Burns JM (2015). Dose-Response of Aerobic Exercise on Cognition: A Community-Based Pilot Randomized Controlled Trial. PLOS ONE.

[CR83] Wells JCK (2012). Ethnic variability in adiposity, thrifty phenotypes and cardiometabolic risk: Addressing the full range of ethnicity, including those of mixed ethnicity: Ethnicity, body composition and cardiometabolic risk. Obesity Reviews.

[CR84] Wright NC, Saag KG, Curtis JR, Smith WK, Kilgore ML, Morrisey MA, Yun H, Zhang J, Delzell ES (2012). Recent trends in hip fracture rates by race/ethnicity among older US adults. Journal of Bone and Mineral Research.

[CR85] Xiao Z, Guo B, Gong J, Tang Y, Shang J, Cheng Y, Xu H (2017). Sex- and age-specific percentiles of body composition indices for Chinese adults using dual-energy X-ray absorptiometry. European Journal of Nutrition.

